# Public Perceptions of the Food and Drug Administration's Regulatory Authority Over Synthetic Nicotine on Twitter: Observational Study

**DOI:** 10.2196/56371

**Published:** 2024-09-19

**Authors:** Jonathan Zou, Juan Ramon Feliciano, Zidian Xie, Dongmei Li

**Affiliations:** 1 Goergen Institute for Data Science University of Rochester Rochester, NY United States; 2 Department of Computer Science University of Rochester Rochester, NY United States; 3 Department of Clinical and Translational Research University of Rochester Medical Center Rochester, NY United States

**Keywords:** FDA, synthetic nicotine, omnibus, Twitter, Food and Drug Administration

## Abstract

**Background:**

The Omnibus Budget Bill, known as H. R. 2471, passed through Congress on March 10, 2022, and was eventually signed by President Biden on March 15, 2022. This bill amended the Federal Food, Drug, and Cosmetic Act granting the Food and Drug Administration (FDA) regulatory authority over synthetic nicotine.

**Objective:**

This study aims to examine the public perceptions of the Omnibus Bill that regulates synthetic nicotine products as tobacco products on Twitter (rebranded as X).

**Methods:**

Through the X streaming application programming interface, we collected and identified 964 tweets related to the Omnibus Bill on synthetic nicotine between March 8, 2022, and April 13, 2022. The longitudinal trend was used to examine the discussions related to the bill over time. An inductive method was used for the content analysis of related tweets. By hand-coding 200 randomly selected tweets by 2 human coders respectively with high interrater reliability, the codebook was developed for relevance, major topics, and attitude to the bill, which was used to single-code the rest of the tweets.

**Results:**

Between March 8, 2022, and April 13, 2022, we identified 964 tweets related to the Omnibus Bill regulating synthetic nicotine. Our longitudinal trend analysis showed a spike in the number of tweets related to the bill during the immediate period following the bill’s introduction, with roughly half of the tweets identified being posted between March 8 and 11, 2022. A majority of the tweets (497/964, 51.56%) had a negative sentiment toward the bill, while a much smaller percentage of tweets (164/964, 17.01%) had a positive sentiment toward the bill. Around 31.43% (303/964) of all tweets were categorized as objective news or questions about the bill. The most popular topic for opposing the bill was users believing that this bill would lead users back to smoking (145/497, 29.18%), followed by negative implications for small vape businesses (122/497, 24.55%) and government or FDA mistrust (94/497, 18.91%). The most popular topic for supporting the bill was that this bill would take a dangerous tobacco product targeted at teens off the market (94/164, 57.32%).

**Conclusions:**

We observed a more negative sentiment toward the bill on X, largely due to users believing it would lead users back to smoking and negatively impact small vape businesses. This study provides insight into public perceptions and discussions of this bill on X and adds valuable information for future regulations on alternative nicotine products.

## Introduction

In May 2016, the Food and Drug Administration (FDA) finalized a “deeming rule” to start regulating electronic cigarettes (e-cigarettes) that contain nicotine “made or derived from tobacco” [1]. Nicotine is an addictive drug and has a powerful effect on human health. It can activate the nicotine-specific receptors in the brain, activating the dopaminergic brain rewarding system and further changing the brain’s structure by increasing the number of nicotine-specific receptors [2]. E-cigarettes with colorful packaging and appealing flavors lure youth into using them. The Centers for Disease Control and Prevention found that e-cigarette use amongst high schoolers increased from 11.7% to 20.8% from 2017 to 2018 [3]. Furthermore, a recent review study has found that adolescent nicotine exposure could lead to subsequent drug abuse by influencing long-term molecular, chemical, and functional changes in the brain [4]. It also affects them by disrupting the growth of brain circuits that control attention and learning [5]. In 2022, it was reported that 16.5% of high school students and 4.5% of middle school students used tobacco products in the past 30 days, with a majority of the products being e-cigarettes [6].

The slight decrease in e-cigarette usage could be attributed to legislation put in place. In 2020, the FDA announced and implemented the e-cigarette flavor enforcement policy to regulate flavored e-cigarettes [1]. As only tobacco-derived e-cigarettes were subject to this regulation, some companies transitioned to selling flavored e-cigarettes containing synthetic nicotine, which is developed in a laboratory, as opposed to tobacco-derived nicotine, to circumvent the FDA regulation and continue the sale of attractive flavored products such as “blueberry ice” and “lychee ice” [7]. Puff Bar, one of the top vape brands among youth [8], markets the idea of “tobacco-free nicotine” as “dramatically improv(ing) flavor while still maintaining the same satisfaction smokers are seeking from their nicotine.” Another product that has transitioned to using synthetic nicotine is oral nicotine pouches. They have become a popular e-cigarette alternative as it is smokeless and can be discreetly used. They come in similar flavors to e-cigarettes and have varying nicotine concentrations [9].

On March 10, 2022, the US Senate passed an Omnibus Budget bill, under which the Consolidated Appropriations Act (H.R. 2471) amended the Federal Food, Drug, and Cosmetic Act granting FDA regulatory authority over synthetic nicotine [10]. The bill had been passed by the US House the day before. President Joe Biden signed the Omnibus Budget bill into law on March 15, 2022 [11], and the law took effect on April 14, 2022 [10]. This legislation broadened the definition of a “tobacco product” to include products “containing nicotine from any source” [10]. Under this legislation, any synthetic nicotine product on the US market could remain on the market until May 14, 2022. By that date, a premarket tobacco product application of those synthetic nicotine products must be submitted to the FDA or the product should come off the market after July 13, 2022 [10].

The use of social media has been on a meteoric rise in the past couple of decades. In 2005, just 5% of Americans were on a social media platform; in 2021, that number went up to 72% [12]. Users can voice their opinions and find discussions on various topics conveniently from their phones. One popular social media platform in the United States is Twitter (rebranded and hereinafter referred to as X), known for its quick, short posts known as “tweets,” users can repost other users’' tweets, known as “retweets.” X had 229 million daily active users in the first quarter of 2022 [13]. X has been used to investigate the public perceptions of tobacco products [14] and tobacco product regulatory policies [15,16], including the FDA flavor ban and the New York State ban on flavored e-cigarettes. According to a recent study, X users were more likely to express a negative attitude than a positive attitude toward the FDA’s approval of Vuse (an e-cigarette brand) [17]. However, regarding the FDA’s proposed menthol cigarette rules, more X users showed a positive attitude rather than a negative one [18]. When comparing X posts between the United Kingdom and Australia, it was found that while a negative attitude toward the policy of e-cigarettes as medical products was prevalent in both countries, there were notable differences in public concerns between the 2 countries [19]. In this study, using X data, we aim to examine the public perceptions of the synthetic nicotine regulation contained in H. R. 2471, specifically user sentiments toward the bill and major topics discussed. Our findings will provide insights into how social media users responded to the bill and future products that could become popular due to the lack of FDA regulation.

## Methods

### Data Collection

Through the X streaming application programming interface, X posts (known as tweets) related to nicotine and cigarettes were collected using relevant keywords, such as “cig*,” “smok*,” “tobacco,” and “nicotine.” To identify tweets relevant to the Omnibus Bill, we further filtered using specific keywords and phrases related to synthetic nicotine and the bill including “tobacco-free nicotine,” “tobacco free nicotine,” “synthetic nicotine,” “synthetic,” “synthetic s-nicotine,” “r-nicotine,” “non-tobacco-derived nicotine,” “tfn,” “omnibus,” “congress,” “senate,” and “bill.” As we were interested in the initial reaction to the passing of the bill, we only considered tweets dated between March 8, 2022, and April 13, 2022. Duplicate tweets were then removed, resulting in a data set containing 1576 tweets. To further ensure the relevance of tweets regarding the federal action toward synthetic nicotine specifically, we manually annotated each tweet and identified 964 relevant tweets.

### Content Analysis

By examining the X data set, 2 authors (JZ and JF) classified each tweet as having a positive, negative, or neutral sentiment toward the bill. Positive posts had a clear attitude in support of the bill, negative posts had a clear attitude against the bill, and neutral posts generally consisted of questions or statements about the bill. Within each sentiment, we classified each tweet into several topics that could further explain the sentiment. Each tweet was assigned only 1 topic and sentiment.

For hand-coding, 200 tweets have been randomly selected from 964 relevant tweets. JZ and JF manually labeled each tweet, respectively, and achieved a notably high agreement rate across topics (κ=0.85) and sentiment (κ=0.90). Any discrepancy between the 2 coders was resolved by a group of 4 members (JZ, JF, ZX, and DL), and the codebook was further developed (Table 1). The topics with positive sentiment toward the bill were “positive implication for limiting teen usage,” which praised the bill for taking away a product targeted toward a younger audience, and “miscellaneous positive public health implications.” There were two categories with a neutral sentiment toward the bill. The first category was “news releases and general facts about the bill”, which contained tweets with news headlines regarding the bill. The second category was “questions relating to the bill,” which were questions regarding the bill such as timeline of enforcement and what it meant for current synthetic nicotine users. There were 4 categories with negative sentiment toward the bill. The first category was “loss of smoking cessation tool,” which criticized the bill as it would now drive users back to what they believe is a more dangerous tobacco product—cigarettes. The second category was “negative implications for small vape businesses,” as the small smoke shops lost a key product that drove business. The third category was “government or FDA mistrust,” as users did not believe the FDA should be regulating these products and that there was a partnership between the government and big tobacco companies. The last category was “call to promote opposition against the bill,” which were tweets calling for people to text senators to show opposition. Based on the codebook developed, the remaining 764 tweets were single-coded by 2 coders (JZ and JF).

**Table 1:** Attitudes and topics in tweets related to the Omnibus Bill on synthetic nicotine from March 8, 2022, to April 13, 2022.

Sentiment and Topics		Count, n (%)	Example post
**Positive (n=164)**			
	Positive implication for limiting teen usage	94 (57.32)	Finally, a step towards reducing nicotine products that target teens. A draft of a government spending bill obtained includes new language that would give the FDA^a^ explicit authority to regulate so-called synthetic nicotine products.
	Miscellaneous positive public health implications	32 (19.51)	Huge policy victory for American health! Congress moves to give powers over synthetic nicotine used in e-cigarettes popular with young people. Part of a massive government spending bill expected to win congressional approval in coming days. #endtobacco
	No specific reason or other	38 (23.17)	We joined in urging Congress to include language in the #omnibus that would give the FDA authority to regulate products such as synthetic nicotine. Happy to report success!
**Neutral (n=303)**			
	News releases and general facts about the bill	268 (88.45)	Bill Granting FDA Regulatory Power Over Synthetic Nicotine Passes
	Questions relating to the bill	35 (11.55)	What Does the Synthetic Nicotine Law Mean for Vapers?
**Negative (n=497)**			
	Loss of smoking cessation tool	145 (29.18)	Yeah and they hid language in that bill that outlaws synthetic nicotine. Millions of lives have been saved with synthetic nicotine. This will drive many to go back to smoking cigarettes
	Negative implications for small vape businesses	122 (24.55)	The FDA lied to small businesses! Please do not pass synthetic nicotine in #omnibus bill, it will destroy the small vapor industry in America! #SmallBusinessMatters
	No specific or other reason opposing the bill	59 (11.87)	Unfortunately, this included giving FDA regulatory power over synthetic nicotine. We may have lost this battle, but the war isn't over. Stay tuned. . . .
	Government or FDA mistrust	94 (18.91)	you see this last bs bill they’re about to pass. Lumping synthetics with tobacco and handing it over to the crooks at the fda?
	Call to promote opposition against bill	77 (15.49)	Send a message to your representative to oppose any ban on synthetic nicotine.

^a^FDA: Food and Drug Administration.

### Ethical Considerations

This study has been reviewed and approved by the Office for Human Subject Protection Research Subjects Review Board at the University of Rochester study ID (STUDY00006570). All tweets have been deidentified and X users are anonymous for this secondary analysis of publicly available X data.

## Results

### Longitudinal Trends in Discussing the Omnibus Bill on X

**Figure 1:**
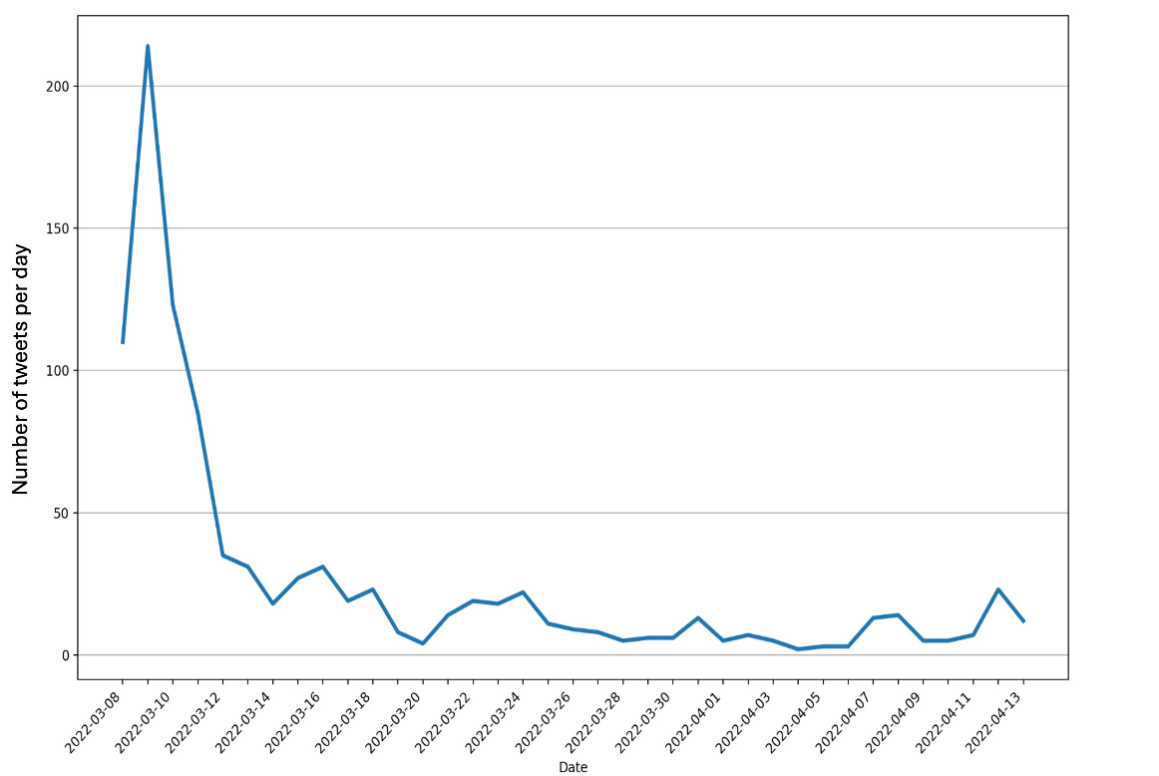
Number of tweets related to the omnibus bill on synthetic nicotine over time.

As shown in Figure 1, roughly 55% (533/964) of the tweets in the data set were posted between March 8, 2022, and March 11, 2022, with an obvious peak (214 tweets) occurring on March 9, 2022. Following the initial spike, there was a general decline in the number of tweets per day from March 11, 2022, to April 13, 2022.

### Public Perception and Discussion of the Omnibus Bill on X

To understand the major topics and sentiments of discussions regarding the Omnibus bill about synthetic nicotine on X, we first categorized each tweet into either a positive, negative, or neutral attitude toward the bill. Among them, 17.01% (164/964) of tweets expressed a positive attitude toward the bill, 31.43% (303/964) of tweets expressed a neutral attitude toward the bill, and 51.56% (497/964) of tweets expressed a negative attitude toward the bill. Based on the results from the 2-proportional z test, the proportion of tweets with a negative attitude toward the bill was significantly higher than those with a positive attitude toward the bill (*P*<.001).

As shown in Table 1, among tweets with a positive attitude toward the bill, the most popular topic was that it is a positive step toward limiting teen usage of synthetic nicotine (94/164, 57.32%), followed by miscellaneous positive public health implications that the bill could bring (32/164, 19.51%). The remaining tweets with a positive attitude toward the bill had no specific reason for that attitude that could be identified (38/164, 23.17%). Of topics with a negative sentiment toward the bill, the most frequently mentioned topic was that banning synthetic nicotine products results in the loss of a smoking cessation tool for those trying to quit smoking (145/497, 29.18%), followed by the negative implications of the ban for small tobacco businesses (122/497, 24.54%). Other notable topics mentioned in tweets with a negative attitude toward the bill were government and FDA mistrust (94/497, 18.91%) and calls for other users to oppose the bill (77/497, 15.49%). Most neutral posts contained content in the form of news releases and general facts about the bill (268/303, 88.45%), and the remainder came in the form of questions about the bill (35/303, 11.55%).

## Discussion

### Principal Findings

This study explored the initial public reaction on X to the passing of H. R. 2471 in the context of the bill’s reclassification of synthetic nicotine as a tobacco product. By mining X data and performing topic and sentiment analysis on 964 tweets posted between March 8, 2022, and April 13, 2022, we showed that over half of the posts had a negative attitude in the context of the reclassification of synthetic nicotine. The most popular reasons for opposition to the bill were that it would drive synthetic nicotine users back to smoking and have negative economic implications for small vape businesses. In addition, about 17.01% (164/964) of tweets had a positive attitude toward the bill in the context of synthetic nicotine, among which the largest topic was that it would limit adolescent usage of nicotine products.

### Comparison With Previous Work

Since this policy is relatively new and synthetic nicotine is also a new product, there are limited previous studies on this topic. This study showed that over half (497/964) of the tweets had a negative sentiment toward the bill. It is reasonable that there is more opposition to the bill, as these social media users are likely users of synthetic nicotine and would be affected by the legislation. People are more inclined to comment on things that negatively affect them than positively. A previous study investigating the public perceptions on X of the FDA flavor ban that occurred in January 2020 showed that there was a significantly higher proportion of negative tweets (63.7%) compared with positive tweets (24.5%) about the FDA policy between the announcement and implementation of the policy [16]. In contrast, another study on the perception of the menthol cigarette ban on X found that 22.82% of tweets had a negative attitude toward the ban, while 27.61% had a positive attitude toward the ban [18]. Modern oral nicotine products with synthetic nicotine are emerging noncombustible nicotine products in recent years [20]. A recent study analyzed the perception of oral nicotine pouches on Reddit (Advance Publications) and showed that 54.2% of Reddit posts had a positive sentiment toward the products, while 15.5% had a negative sentiment [14]. While we cannot directly determine if users had a positive or negative sentiment toward synthetic nicotine, we could infer that users with a positive attitude toward oral nicotine products are more likely to have a negative sentiment toward the bill regulating synthetic nicotine.

The main reason for supporting the Omnibus bill on X was that it would improve public health as another tobacco product would be off the market. A subset of users thought it was beneficial, especially to young adults and teens, as they were more susceptible to getting hooked on the product. The topics we found were similar to a study on whether the NY State flavor ban affected the public sentiment about e-cigarettes on X. In that study, the main topic for tweets with negative sentiments toward e-cigarettes was how it drove teen vaping [15]. Another point of emphasis on synthetic nicotine is how it is marketed. A main synthetic nicotine product called Puffbar comes in bright and colorful packaging that looks relatively harmless [8]. A recent study goes in-depth into synthetic nicotine companies’ different marketing strategies [21]. They found that tobacco companies leverage this product as “cleaner” and “safer” as well as “improved taste” to deflect attention away from its main purpose, which is regulatory escape. Companies even alter the mandated FDA nicotine addiction warning by swapping “nicotine” for “synthetic nicotine” or “tobacco-free nicotine” to imply a lower risk of addiction. Furthermore, synthetic nicotine has been able to have success in e-commerce companies because they evade screenings that would usually pick up tobacco products. These findings are important because they show how tobacco companies will misleadingly market synthetic nicotine even though little is known about its health effects. The marketing strategies of synthetic nicotine will likely impact teens more, which explains why the faction of tweets supports the synthetic nicotine bill because it will limit the teen usage of the product. This aligns with the Truth Initiatives’ main findings on synthetic nicotine as they cite misleading marketing directed at younger users as to why the product is harmful [8].

There were 3 main reasons for opposing the Omnibus bill on synthetic nicotine. First, social media users believe that more users will return to smoking without synthetic nicotine, which is a more harmful tobacco product. Increasing scientific evidence showed the benefits of completely switching to e-cigarettes from smoking for current smokers [22,23]. The second reason is that this bill will benefit big businesses and hurt small businesses. They believe many small vape shops will need to shut down as more users return to smoking, driving more money into big tobacco companies. These companies are also more closely regulated by the FDA, so that the FDA will have more control over tobacco legislation. The last reason is that users believe that the government or FDA should not have control over synthetic nicotine legislation as they are not looking out for the health of Americans. The reasons are all aligned to tell a story of why users are opposed to this bill. With most tweets having a negative sentiment toward the bill, enforcement and compliance challenges need to be considered. Psychological reactance may lead users to search for alternative ways to obtain synthetic nicotine aggressively. Underground markets for synthetic nicotine may begin to rise, and those present dangers as the products are unregulated. Our findings line up quite similarly compared with previous work on FDA flavor enforcement. A previous study also suggested that X users began discussing alternative ways to get e-cigarettes after the FDA e-cigarette flavor enforcement policy was put in place [16]. That certainly aligns with the growth in the popularity of synthetic nicotine and Puff Bars. Our data provide insights into what users are discussing regarding alternatives to synthetic nicotine. Some tweets referenced the rise of cannabis products, such as edibles, and that those could be next for the users to pick up. Edibles can come in unassuming forms, which could become popular amongst many users such as teens. The FDA does not yet regulate these products. Furthermore, this legislation could lead to the introduction of similar, but unbanned alternatives such as nicotine analog and betel nuts to the market, as was the case with the introduction of clove cigars following the ban of flavored cigarettes in the United States [21,24].

The longitudinal trend showed that the discussion about the bill was amplified when the Congress and Senate voted on the bill. The first 4 days of data collection have the highest daily tweet counts. The “call to promote opposition against the bill” topic had the largest proportion of the total tweets during the first 4-day period. Of the 76 tweets on the topic, 67 were in the first 4 days, indicating a strong push on X in opposition to the bill in the days leading up to and during the bill going through the Congress. The increase in tweets during the legislation stage of a tobacco policy was also found during the FDA e-cigarette flavor enforcement policy. The previous study on the FDA e-cigarette flavor enforcement policy showed that the highest number of daily tweets occurred between the announcement and implementation of the policy [16]. These results indicate that social media is valuable in promoting action when legislation is announced and voted on. The prevalence of news releases about the bill could also contribute to the increase in posts and drive discussion during that time.

### Limitations

There are several limitations to this study. First, we did not collect the geolocation information from each tweet, which could be used to analyze the sentiment and topics based on states to see which states had more favorable or unfavorable views on the bill. Second, we could not determine the demographics (such as race, gender, and age) of X users with different attitudes toward the bill, which might help us understand how different population responded to this bill. Third, X data cannot be generalized to the entire population since the demographic composition of X users is slightly different from the whole population. X users often represent the loud minority on a topic as they want their voices to be heard. Therefore, they do not represent the whole population. Fourth, with the fast pace of social media, X discussions can be dynamic, and sentiment toward the bill may shift over time if there are new developments. Finally, the sample size was relatively small, which might create some biases. Given the high concentration of tweets during the first 4 days of the study period, no longitudinal trend analysis on sentiment was performed.

### Conclusions

By examining the topics and sentiment of tweets related to the Omnibus bill on synthetic nicotine, we showed that a majority of tweets had a negative sentiment toward the bill, mainly because it would drive users back to smoking. The findings from this study provide insights into the public reaction to the bill and can be used for future government policy toward tobacco products.

## Abbreviations

FDA: Food and Drug Administration

## Data Availability

The data presented in this study can be requested from the corresponding author.

## References

[1] (2020). FDA finalizes enforcement policy on unauthorized flavored cartridge-based e-cigarettes that appeal to children, including fruit and mint.

[2] Bishop S (2012). How Do Smoker’s Brains Change in Response to High Nicotine Levels?.

[3] (2019). Tobacco use by youth is rising.

[4] Ren M, Lotfipour S (2019). Nicotine gateway effects on adolescent substance use. West J Emerg Med.

[5] Majmundar A, Xue Z, Asare S, Nargis N (2023). Trends in public interest in shopping and point-of-sales of JUUL and Puff Bar 2019-2021. Tob Control.

[6] (2022). Results from the annual national youth tobacco survey.

[7] Puff Bar.

[8] (2022). What is synthetic nicotine and what does it mean for the youth vaping epidemic?.

[9] (2024). What is Zyn and what are oral nicotine pouches?.

[10] (2022). H.R.2471 - Consolidated Appropriations Act, 2022.

[11] (2022). Bill signed: H.R. 2471.

[12] (2024). Social media fact sheet.

[13] Dixon S (2022). Number of monetizable daily active X (formerly Twitter) users (mDAU) worldwide from 1st quarter 2017 to 2nd quarter 2022 (in millions).

[14] Shao Y, Zou J, Xie Z, Mayne RG, Ossip DJ, Rahman I, McIntosh S, Li D (2022). Perceptions of oral nicotine pouches on reddit: observational study. J Med Internet Res.

[15] Gao Y, Xie Z, Li D (2022). Investigating the impact of the New York State flavor ban on e-Cigarette-related discussions on Twitter: observational study. JMIR Public Health Surveill.

[16] Lu X, Sun L, Xie Z, Li D (2022). Perception of the food and drug administration electronic cigarette flavor enforcement policy on Twitter: observational study. JMIR Public Health Surveill.

[17] Lee S, Xie Z, Xu E, Shao Y, Ossip DJ, Li D (2023). Public perceptions of the FDA's marketing authorization of vuse on Twitter/X. Front Public Health.

[18] Zhou R, Tang Q, Xie Z, Li D (2023). Public perceptions of the food and drug administration's proposed rules prohibiting menthol cigarettes on Twitter: observational study. JMIR Form Res.

[19] Lou X, Liu P, Xie Z, Li D (2023). Public perceptions on the policy of electronic cigarettes as medical products on Twitter. Int J Environ Res Public Health.

[20] Felicione NJ, Schneller LM, Goniewicz ML, Hyland AJ, Cummings KM, Bansal-Travers M, Fong GT, O'Connor RJ (2022). Oral nicotine product awareness and use among people who smoke and vape in the U.S. Am J Prev Med.

[21] Ramamurthi D, Chau C, Lu Z, Rughoobur I, Sanaie K, Krishna P, Jackler RK (2022). Marketing of “Tobacco-Free” and “Synthetic Nicotine” products.

[22] Münzel T, Kuntic M, Steven S, Hahad O, Daiber A (2020). Is vaping better than smoking cigarettes?. Eur Heart J.

[23] Marques P, Piqueras L, Sanz MJ (2021). An updated overview of e-cigarette impact on human health. Respir Res.

[24] Delnevo CD, Hrywna M (2015). Clove cigar sales following the US flavoured cigarette ban. Tob Control.

